# Quantitative myocardial inflammation assessed using a novel USPIO-Magnetic Resonance Imaging acquisition and analysis protocol

**DOI:** 10.1186/1532-429X-15-S1-O114

**Published:** 2013-01-30

**Authors:** Scott Semple, Shirjel R Alam, Tom J MacGillivray, Marc R Dweck, Anoop S Shah, Jenny Richards, Chengjia Wang, Ninian Lang, Graham McKillop, Saeed Mirsadraee, Renzo Pessotto, Vipin Zamvar, Peter Henriksen, David Newby

**Affiliations:** 1 Centre for Cardiovascular Research, University of Edinburgh, Edinburgh, UK; 2 Clinical Research Imaging Centre, University of Edinburgh, Edinburgh, UK; 3 Department of Cardiology, Royal Infirmary of Edinburgh, Edinburgh, UK; 4 Department of Vascular Surgery, Royal Infirmary of Edinburgh, Edinburgh, UK; 5 Department of Radiology, Royal Infirmary of Edinburgh, Edinburgh, UK; 6 Department of Cardiac Surgery, Royal Infirmary of Edinburgh, Edinburgh, UK

## Background

Ultrasmall superparamagnetic particles of iron-oxide (SUPIO) particles can be used as a magnetic resonance imaging contrast (MRI) agent. Due to their dextran coating and small diameter (<30 nm), they are phagocytised by inflammatory cells [1].

The aim of this study was to assess whether a novel acquisition/registration USPIO-MRI protocol could be used to assess myocardial cellular inflammation. Myocardial infarction and cardiac surgery was used to model myocardial inflammation [2]. An influx of macrophages can be seen in post-mortem histology, however the dynamics of in vivo patho-physiology is uncertain [3].

## Methods

16 patients underwent MRI 2-4 days after ST-elevation myocardial infarction (STEMI) at baseline and 24-hours after intravenous USPIO infusion (4 mg/kg; Ferumoxytol, AMAG; n=10) or no infusion (n=6). Between 5 and 28-days following on-pump coronary artery bypass graft (CABG) surgery, 32 patients underwent the same protocol with all patients receiving USPIO.

3T-MRI (Siemens Medical) was performed to optimise USPIO sensitivity. Baseline and 24-hour multi-echo T2*-weighted sequences were spatially registered to generate an R2* (1/T2*) map to assess USPIO uptake. R2* maps were spatially registered using custom software (Matlab/Analyze) to late gadolinium enhancement (LGE) images to confirm USPIO localisation in the infarct zone. CABG R2* maps were analysed using a standard 17-segment model

Data comparing CABG to MI cohorts was analysed by one-way and repeated measures ANOVA with Dunn's post-test. Data comparing change in R2* values within cohorts was analysed by Wilcoxon matched pairs test.

## Results

Consistent with reticuloendothelial uptake, R2* values increased in the liver & spleen following USPIO administration. In the myocardial infarct, there was a large R2* increase from 41.0±12.0 (baseline) to 155±45.0 s-1 (p<0.001) at 24h. A lower magnitude response was seen in the remote myocardium from 39±3.2 to 80±14.9 s-1 (p<0.002), consistent with myocardial inflammation that occurs post infarction [4[.

In CABG patients the R2* signal increased: 45±7s-1 to 118± 22 s-1 at 24h (p<0001). The increase in myocardial R2* values was greater than in the remote myocardium of patients with STEMI, but lower than the infarct zone itself (p<0.001 and p<0.05 respectively).

## Conclusions

USPIO are taken up into inflamed myocardial tissue and can be quantitatively assessed using a multi-parameter MRI acquisition protocol with optimised spatial registration analysis based on a custom mutual information algorithm.

This represents a novel imaging technique of assessing myocardial inflammation, as well as providing a useful research tool in investigating tissue inflammation.

## Funding

Funding was provided by the Medical Research Council, Chest Heart & Stroke Scotland, the British Heart Foundation and the Scottish Universities Physics Alliance.

The Welcome Trust Clinical Research Facility and the Clinical Research Imaging Centre are supported by NHS Research Scotland (NRS) through NHS Lothian.

**Figure 1:**
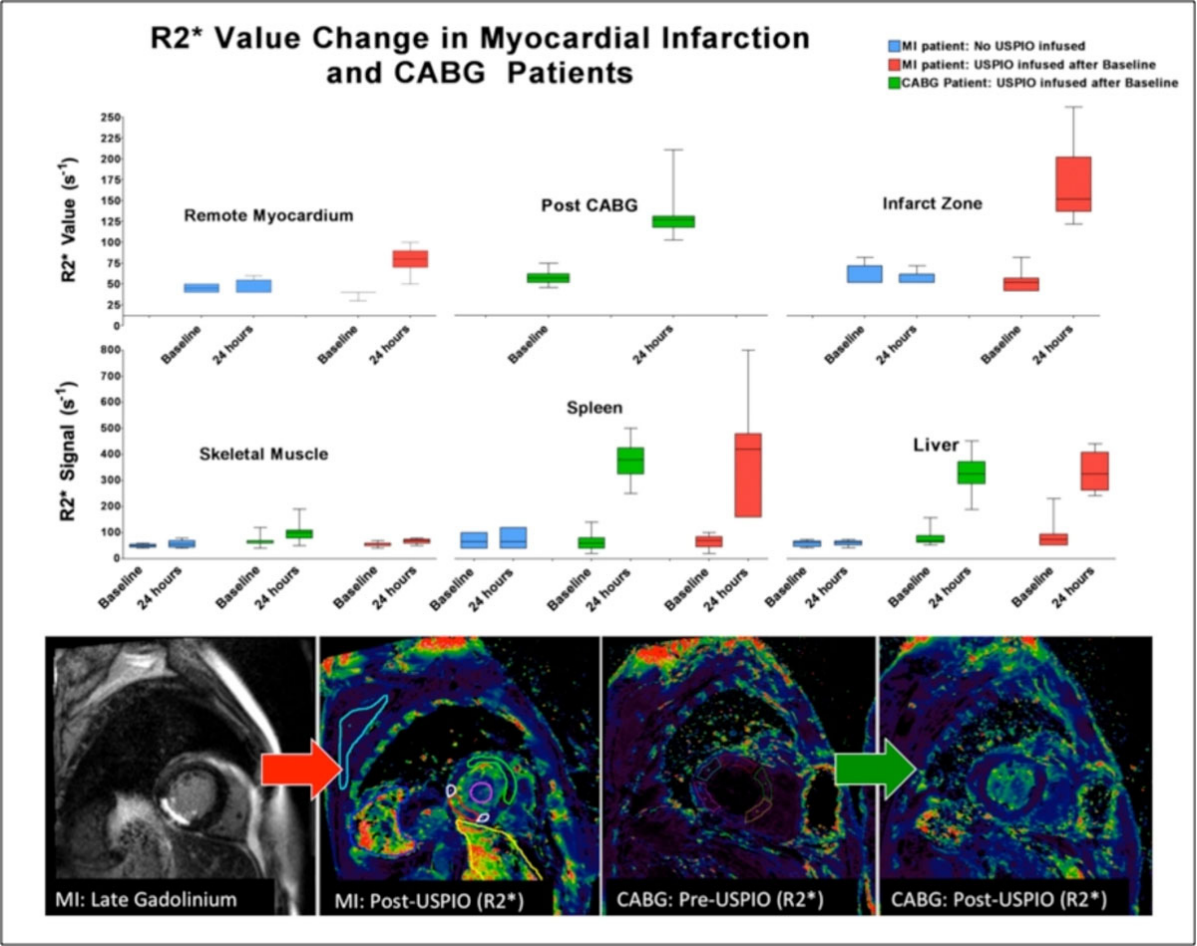
Increase in R2* values in control STEMI patients (no USPIO) (blue, n=6), STEMI patients given USPIO (red, n=10) and CABG patients (green, n=32). Most significant R2* increase (and hence myocardial inflammation) observed in infarct zones, with CABG myocardial R2* increase between infarct and myocardium remote to infarction. The spatially registered regions of interests (ROI) for quantifying R2* value for STEMI and CABG patients are shown in the MRI images at the bottom of the figure.

